# Calcium and linoleic acid supplements in the prevention of preeclampsia

**Published:** 2016-03-30

**Authors:** Leonelo E Bautista

Alzate et al. 1 http://www.ncbi.nlm.nih.gov/pmc/articles/PMC4732504/, conducted a nested case-control study to (quote) "estimate the protective effect from calcium [supplement] alone [CC], compared to calcium plus conjugated linoleic acid [CC+CLA] in nulliparous women at risk of preeclampsia". Based on a crude analysis of the data in Table 3 1, they concluded that neither CC nor CC+CLA reduced the risk of preeclampsia in the whole sample, but that CC+CLA significantly decreased risk among women 13-18 years old. A quick look analysis of the data in this table shows this conclusion is mostly based on the fact that none of the cases in 13-18 year old women was treated with CC+CLA. Contrary to the authors' interpretation, this does not point to a protective effect of CC+CLA, it simply indicates that the assumption of positivity has being violated and, consequently, that an effect for this age group cannot be estimated 2. In fact, the probability of getting no treated cases in this age-group was 28%, since only 15.5% of all women received CC+CLA. Also, accurate estimates of effect in women 34-45 years old were not possible, because there were only seven women who used CC+CLA in this age group. In spite of the limited sample size, the authors restricted their attention to the apparent protective effect of CC+CLA in 13-19 year old women, while ignoring apparent harmful effects in older women. I estimated age-specific rate ratios (RR) by fitting a saturated conditional complementary log-log 3 to the data in Table 3 and found that CC+CLA was protective among women 13-19 (RR= 0.61, 95% CI: 0.41- 0.90), but harmful in women 19-34 (RR= 1.74, 95% CI: 1.21- 2.50) and 35-45 years old (RR= 4.98, 95% CI= 1.74-14.30). Of course, this approach is an improvement over a naive crude analysis, but does not solve the problem of violation and near violation of positivity described above. An overall age-adjusted RR was 1.02 (95% CI= 0.89-1.17; p= 0.756). Thus, this study provides no evidence of a beneficial effect of CC+CLA in preventing preeclampsia in any age group.

On the other hand, the authors neglected to explain why the total number of women is 2,703 in Table 3 and 1,441 in Figure 1 and Table 2 1. More important, it is surprising that they restricted their attention to the age-specific effects of CC+CLA, which were obviously unidentifiable, while ignoring the obvious age-related decrease in the effect of CC shown in Table 3: odds ratios of 1.3, 0.9, and 0.4 in 13-18, 19-34, and 35-45 years old, respectively. In fact, corresponding age-specific RR from a clog-log model with a treatment-by-age interaction (p= 0.069) were 1.44 (95% CI= 0.85-2.44), 0.92 (95% CI= 0.73-1.16), and 0.59 (95% CI= 0.34-1.01). This pattern could have resulted not from an effect of CC, but from CC being more frequently prescribed to younger women, who have a higher risk of preeclampsia. This selective use of treatment leads to confounding by indication, a well-recognized limitation of observational studies of the effectiveness of therapeutic interventions 4. Unfortunately, the authors made no attempt to address this type of bias, since they disregarded any clinical factor, such as blood pressure, that could increase the likelihood of both treatment with calcium supplements and risk of preeclampsia.

Moreover, the authors' claim that the beneficial effects of CC+CLA were greater than those of CC is not supported by the data. First, one treatment could not be better than the other because neither of them decreased the risk of preeclampsia. Second, no formal comparison of the two treatments was made. I tested this hypothesis by fitting a saturated clog-log model to the data (Table 3, n= 2,703) and found that none of the treatments decreased the risk of preeclampsia, and that CC (RR= 0.89) seemed more protective than CC+CLA (RR= 1.01), but not significantly so (p= 0.60). Of course, the authors' findings as well as those from my analyses are likely biased, due to the lack of adjustment for confounding factors. Basically, these findings are of no use for clinical or policy decision making.

In spite of very large trials showing no clinical benefits 5,6, calcium supplements are still widely offered to women at high risk of preeclampsia in developing countries 7. Maybe it is time to re-evaluate their usefulness to prevent preeclampsia by looking again at the existing data. But this time with the clear purpose of avoiding confirmation bias 8 and keeping in mind, as Feynman argued, that "the first principle [of science] is that you must not fool yourself-and [yet] you are the easiest person to fool" 9. 
